# Enhanced glycolysis is associated with aggressive tumor phenotype and worse outcomes in hepatocellular carcinoma patients

**DOI:** 10.3389/fmed.2025.1588604

**Published:** 2025-08-26

**Authors:** Masanori Oshi, Colin Rog, Li Yan, Itaru Endo, Kazuaki Takabe

**Affiliations:** ^1^Department of Surgical Oncology, Roswell Park Comprehensive Cancer Center, Buffalo, NY, United States; ^2^Department of Gastroenterological Surgery, Yokohama City University Graduate School of Medicine, Yokohama, Japan; ^3^Department of Biostatistics and Bioinformatics, Roswell Park Comprehensive Cancer Center, Buffalo, NY, United States; ^4^Division of Digestive and General Surgery, Niigata University Graduate School of Medical and Dental Sciences, Niigata, Japan; ^5^Department of Breast Surgery, Fukushima Medical University School of Medicine, Fukushima, Japan; ^6^Department of Surgery, Jacobs School of Medicine and Biomedical Sciences, State University of New York, Buffalo, NY, United States

**Keywords:** biomarker, carcinogenesis, hepatocellular carcinoma, glycolysis, survival

## Abstract

**Background:**

Adenosine Triphosphate (ATP) is mainly generated by oxidative phosphorylation in non-malignant cells, whereas malignant cells rely predominantly on glycolysis for energy production, known as the “Warburg effect.” This study used the gene set variation analysis (GSVA) algorithm to evaluate glycolysis signaling in hepatocellular carcinoma (HCC), and investigated its relationship with tumor aggressiveness and patient prognosis.

**Methods:**

Enhanced level of glycolysis signaling was measured using the “Hallmark-GLYCOLYSIS” gene set in the MSigDB, applied via GSVA across multiple independent HCC cohorts.

**Results:**

There was no significant difference in glycolysis signaling between HCC and other liver diseases in two cohorts. However, enhanced glycolysis signaling linked to gene sets associated with cell proliferation and cancer-promoting pathways, such as unfolded protein response, epithelial mesenchymal transition, and apoptosis, consistently in both cohorts. HCC with high glycolysis signaling score showed increased homologous recombination deficiency (*p* = 0.004), intratumor heterogeneity (*p* = 0.005), and mutation burden (*p* = 0.022). No consistent associations with glycolysis were observed with immune cells infiltration nor cytolytic activity, except for Th1 cells. Clinically, high glycolysis signaling correlated with advanced tumor stage and significantly worse survival outcomes, serving as an independent prognostic biomarker [hazard ratio (HR) = 6.78 and *p* < 0.001 in OS, HR = 6.56 and *p* = 0.027 in DSS].

**Conclusion:**

Elevated glycolysis signaling is linked to enhanced malignant pathways, genomic instability, and worse clinical outcomes in HCC, indicating its potential as a prognostic biomarker.

## 1 Introduction

In non-malignant cells, Adenosine Triphosphate (ATP) produced by mitochondrial oxidative phosphorylation provides most of the energy required for cellular metabolism. In contrast, malignant cancer cells are known to prioritize glycolysis over oxidative phosphorylation for their energy demands (1, 2). Cancer cell energy metabolism is characterized by active glucose uptake and aerobic glycolysis (3). While a seemingly uneconomic method of energy supply, it is required by cancer cells for biosynthesis and continued proliferation. Termed the “Warburg effect,” this feature of cancer cell energy metabolism was first observed by Otto Warburg in the 1920s (4).

Progression and patient survival of hepatocellular carcinoma (HCC) have been linked to the activation of glycolysis pathways (5). Elevated glycolysis activity in tumor cells is correlated with more aggressive cancer phenotypes and worse patient survival. To this end, glycolysis-related marker expressions are expected to serve as prognostic indicators. Currently many studies are focusing on therapies that target glycolytic enzymes and pathways. Specifically, inhibiting pivotal enzymes such as hexokinase 2 (HK2) and pyruvate kinase M2 (PM2) could potentially disrupt the energy supply of cancer cells, thereby hindering tumor growth. Moreover, glycolytic pathway activation is recognized as a contributing factor to treatment resistance. Therefore, combination therapies that target the glycolytic system hold promise for overcoming this resistance and enhancing therapeutic efficacy (6).

To date, numerous attempts have been made to identify a gene expression that reflects the aggressiveness of cancer and be used as a biomarker. For example, we have recently reported that Ki67 gene expression is associated with aggressive phenotype of HCC (7) while Spinster homolog 2 expression correlates with improved HCC patient survival (8). However, the heterogeneity and complexity of tumor biology often limit the predictive power of single-gene approaches. To address this, gene set variation analysis (GSVA) has emerged as a method to capture pathway-level activity across tumors, offering a more integrated view of cancer biology. In our previous studies, we utilized the GSVA method to demonstrate that various gene set pathway scores, such as reactive oxygen species, apical junctions, bile acid metabolism, or E2F targets, are associated with certain clinical outcomes in cancer (9–12). We have also published that BRCAness scoring can serve as a more effective biomarker compared to individual BRCA gene analysis (13). Indeed, we have previously generated a GSVA algorithm to reflect glycolysis activity and found that increased glycolysis in tumor microenvironment was associated with worse survival in triple-negative but not hormone receptor-positive breast cancers (13).

In this study, we applied a previously validated glycolysis pathway score to multiple HCC cohorts to evaluate its clinical relevance, including associations with genomic alterations, tumor immune microenvironment, and patient survival.

## 2 Materials and methods

### 2.1 Acquisition of HCC samples

655 samples with mRNA expression and clinicopathological data were obtained from multiple public databases including the Cancer Genome Atlas (TCGA) (14) and GSE6764 (15), 76427 (16), and 89377 (17) cohorts.

### 2.2 Glycolysis signaling score

Gene Set Variation Analysis (GSVA) is an unsupervised, non-parametric method for estimating variations in gene set enrichment across individual samples in an expression dataset. Unlike traditional gene set enrichment analysis (GSEA), which compares predefined groups of samples, GSVA evaluates each sample on an individual basis. This approach enables researchers to identify changes in pathway activity at the single-sample level, which is particularly valuable for uncovering variable biological processes or pathways in studies of complex diseases such as cancer. GSVA is especially useful in cases where sample classifications are either unavailable or unclear (18, 19). In this study, the enhanced level of glycolysis signaling in individual samples was assessed using the score values calculated by the GSVA algorithm. The gene sets related to glycolysis signaling were identified using the Molecular Signatures Database (MSiGB).

### 2.3 Biological function analysis

A gene set variation analysis (GSEA) algorithm (20) was used to demonstrate the differentiation of biological function of hallmark cancer signaling between low and high inflammatory response signaling HCC within each cohort. Pathways demonstrating significant enrichment were analyzed according to GSEA recommendations with a set false discovery rate (FDR) of <25%. As we have previously reported, the MSigDB gene set collection was utilized to obtain hallmark cancer gene sets (21–25).

### 2.4 Other scores

As previously demonstrated by our group, the cytolytic activity score (CYT) was calculated using the geometric mean of the expression values of granzyme A (GZMA) and perforin (PRF1) measured in transcripts per million (TPM) (26–28). To evaluate the composition of the intra-tumor microenvironment, cellular fractions of 64 unique immune and stromal cell types were estimated by an xCell algorithm as we have previously demonstrated (29–33).

### 2.5 Statistical analysis

The threshold for categorizing low and high glycolysis signaling groups was set at the top quartile of points within each cohort. Kruskal-Wallis or Mann-Whitney U tests were used for comparisons between groups. To examine the relationship between the score and patient survival outcomes, univariate and multivariate Cox regressions were utilized. Statistical analysis and data visualization were executed with R (version 4.1.0) and Microsoft Excel (version 16), with a *p*-value < 0.05 considered statistically significant.

## 3 Results

### 3.1 The glycolysis signaling level was similar in several forms of liver disease including HCC

**FIGURE 1:**
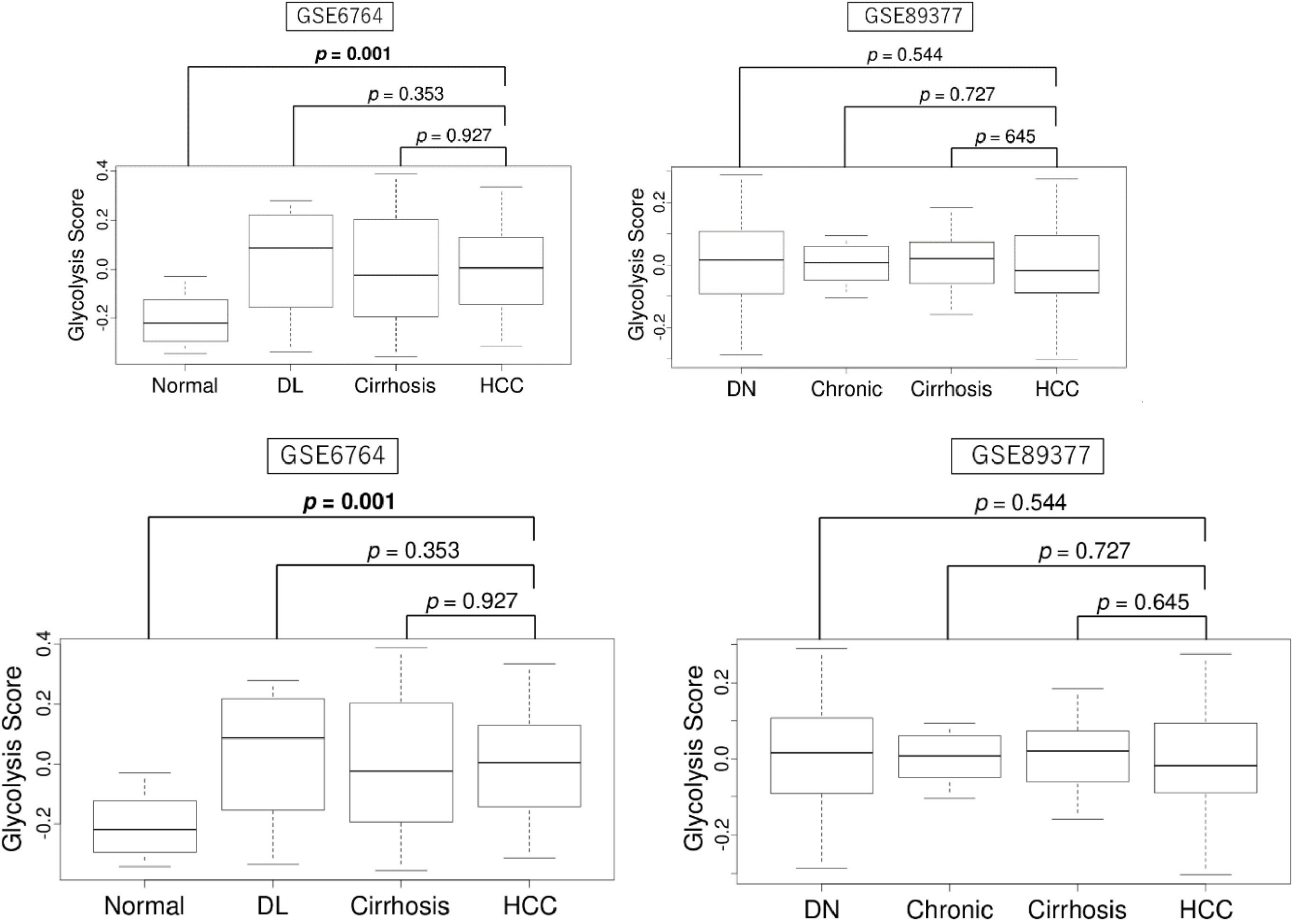
The enhanced level of glycolysis signaling in liver tissues during the carcinogenic sequence of HCC. **(A)** Boxplots of glycolysis signaling score of normal liver tissue, dysplastic nodule (DL), cirrhosis, and HCC (*n* = 10, 17, 13, and 35, respectively) in the GSE6764 cohort and by normal liver tissue, dysplastic nodule (DN), chronic hepatitis, cirrhosis, and HCC (*n* = 13, 22, 20, 12, and 40, respectively) in the GSE89377 cohort. Kruskal-Wallis and Mamm-Whitney U tests were used to investigate for significant differences between groups.

We first investigated the differential levels of enhanced glycolysis signaling in liver tissue samples representative of several forms of liver disease. We found that there were no significant differences in glycolysis signaling score between HCC and other forms of liver disease including dysplastic nodule and cirrhosis, but that significant differences were present between normal tissue and HCC in the GSE6764 cohort (Figure 1). There were also no differences between HCC and other forms of liver disease including dysplastic nodule, chronic hepatitis, and cirrhosis in the GSE89377 cohort.

### 3.2 High levels of glycolysis signaling in HCC enriched cell proliferation-related and other pro-cancerous gene sets

**FIGURE 2:**
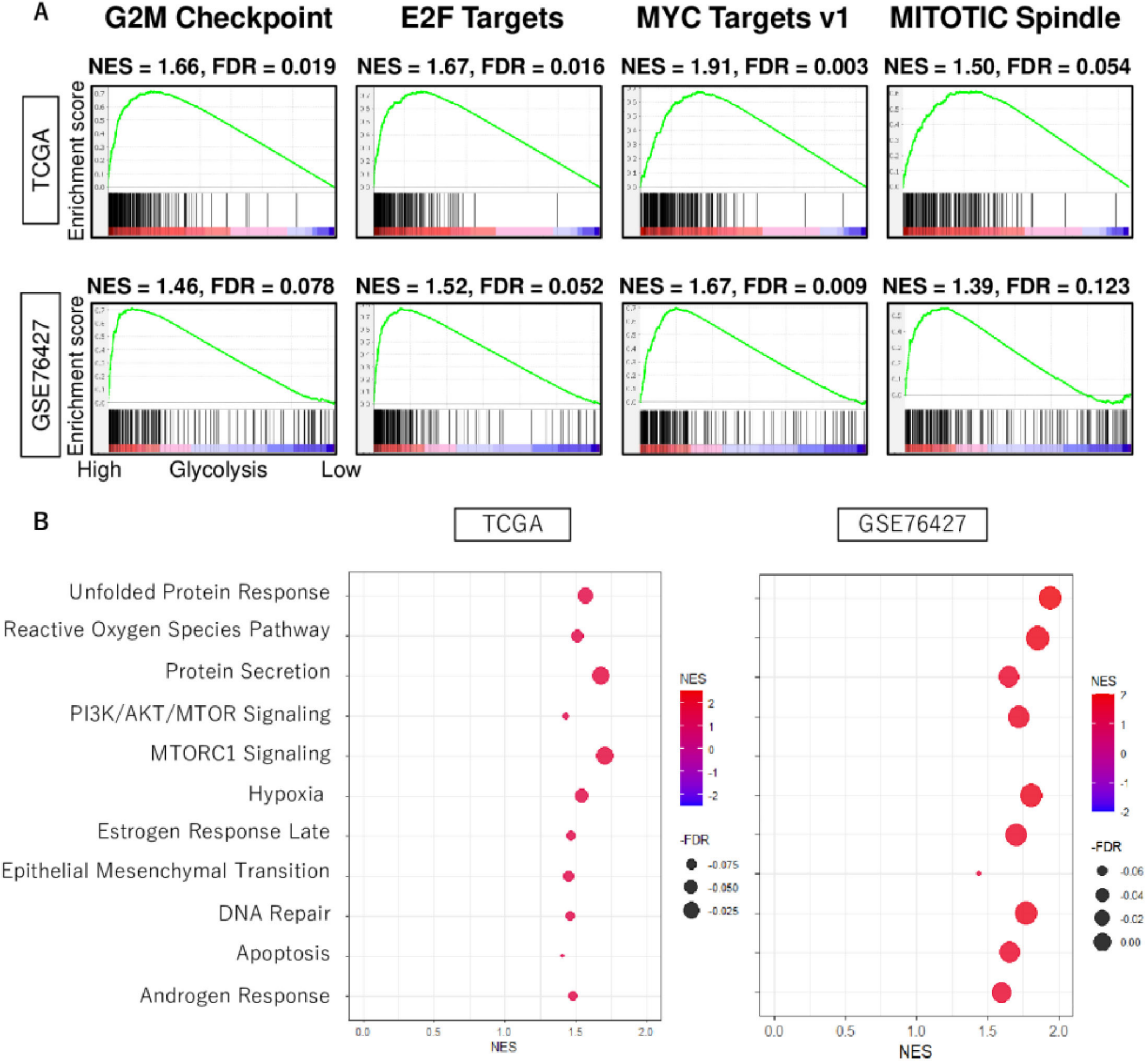
The biological function features of high glycolysis signaling score in HCC. **(A)** Enrichment curves of cell proliferation-related gene sets; G2M checkpoint, E2F targets, MYC target v1, and MITOTIC spindle in TCGA and GSE76427 cohorts. **(B)** Dot plots of unfolded protein response, reactive oxygen species pathway, protein secretion, PI3K/AKT/MTOR signaling, MTORC1 signaling, hypoxia, estrogen response late, epithelial mesenchymal transition, DNA repair, apoptosis, and androgen response, which were enriched significantly in HCC with enhanced glycolysis signaling score consistently in both cohorts. NES, normalized enrichment score; FDR, false discovery rate.

Based on the gene set enrichment analysis algorithm with the MSigDB hallmark gene set collection, the high glycolysis signaling score group was observed to be related to cell proliferation-related gene sets, including G2M checkpoint, E2F targets, MYC targets v1, and MITOTIC spindle consistently in both TCGHA and GSE76427 cohorts (Figure 2A). Furthermore, they also highly enriched other pro-cancerous signaling, including unfolded protein response, reactive oxygen species pathway, protein secretion, PI3K/AKT/MTOR signaling, MTORC1 signaling, hypoxia, estrogen response late, epithelial mesenchymal transition, DNA repair, apoptosis, and androgen response consistently both cohorts (Figure 2B). These results demonstrated that enhanced level of glycolysis signaling was involved in the activity of various hallmark signaling pathways of cancer.

### 3.3 High glycolysis signaling score HCC was significantly associated with high level of homologous recombination defects, intratumor heterogeneity, fraction altered, and mutation rates

**FIGURE 3:**
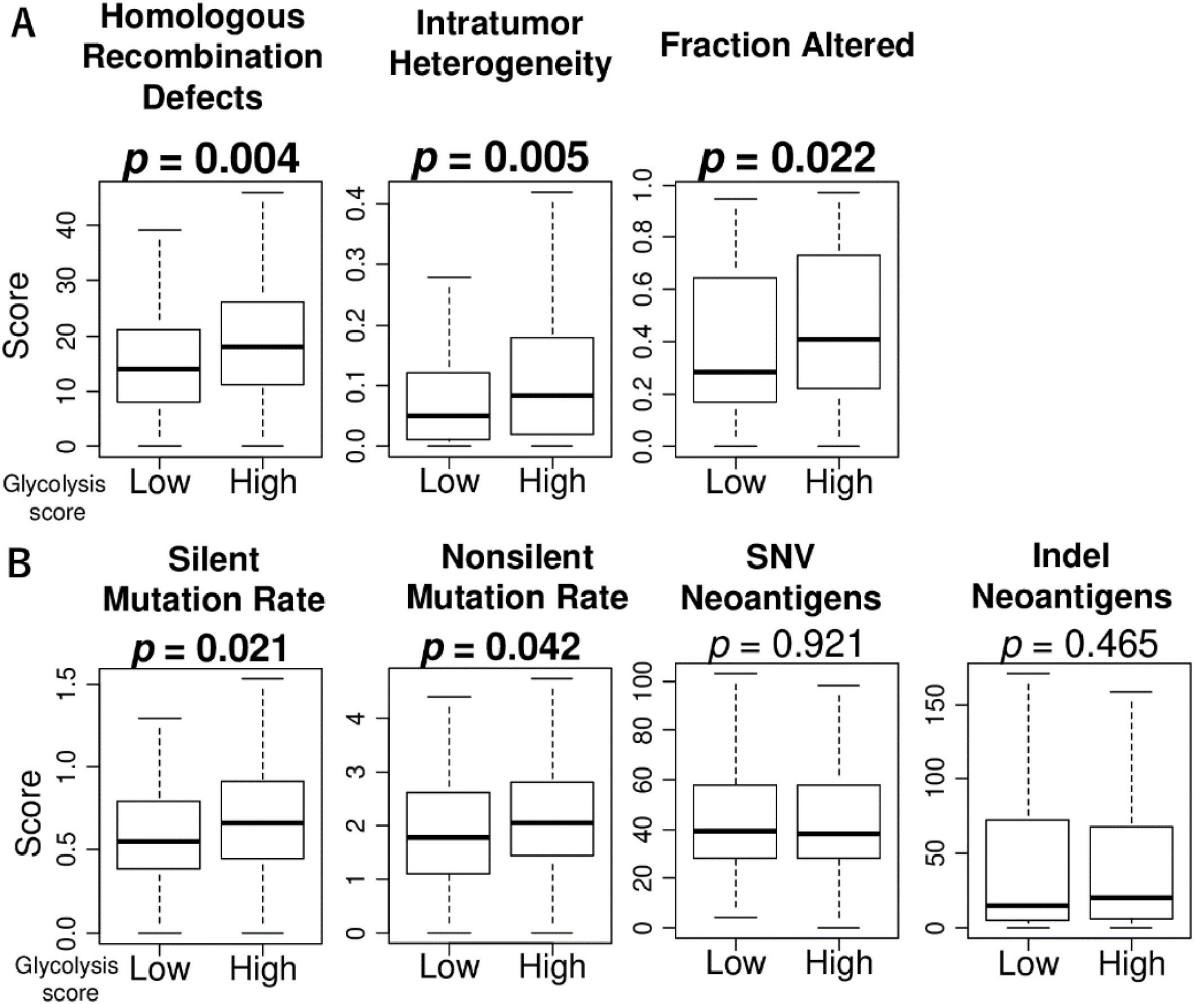
The association of glycolysis signaling with homologous recombination defects, intratumor heterogeneity, fraction altered, as well as mutation-related score in HCC. Box plots of **(A)** homologous recombination defects, intratumor heterogeneity, and fraction altered score level, and **(B)** mutation-related scores; silent mutation rate, non-silent mutation rate, single nucleotide variation neoantigens, and indel neoantigens, by low and high glycolysis score groups in the TCGA cohort. Mann Whitney U tests were used to investigate for significant differences between groups. SNV, single nucleotide variation.

Due to the inherently high heterogeneity of HCC, the association of glycolysis signaling score with intratumor heterogeneity as well as mutational load is of interest. To investigate this association, we used several scores calculated by Thorsson et al. in the TCGA cohort (34). Compared to the low score group, we found that high glycolysis signaling scores were significantly associated with high level of homologous recombination defects, intratumor heterogeneity, and fraction altered, as well as silent and non-silent mutation rates (Figures 3A, B). There were no association between glycolysis signaling score and neoantigen levels. These results suggest that HCC with enhanced glycolysis signaling was significantly associated with higher homologous recombination defects, intratumor heterogeneity, fraction altered, and mutation rates.

### 3.4 High glycolysis signaling HCC was not associated with infiltration fraction rate or function of immune cells in the tumor microenvironment

**FIGURE 4:**
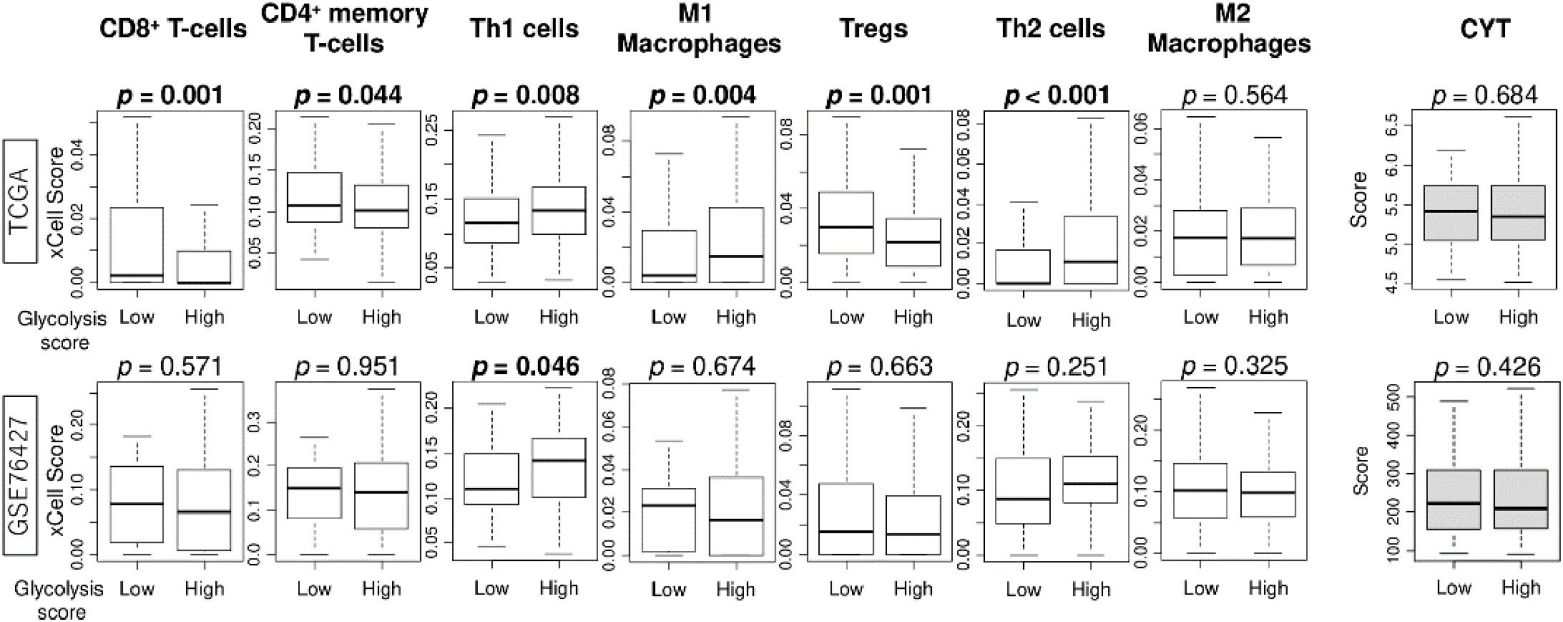
The association of enhanced glycolysis signaling with infiltration rates of immune cells in the tumor microenvironment of HCC in TCGA and GSE76427 cohorts. Boxplots of infiltration rate of CD8 + and CD4 + memory T cells, Th1 cells, M1 macrophages, Tregs, Th2 cells, and M2 macrophages, as well as cytolytic activity score, by low and high glycolysis signaling groups, in both cohorts. Mann Whitney U tests were used to investigate for significant differences between groups. Th, Th1, helper T type 1; Th2, helper T type2; CYT, cytolytic activity.

Since the association between intratumor heterogeneity and tumor immune microenvironment (TIME) of HCC has been reported in recent years (35), we investigated the relationship between glycolysis signaling score levels and the infiltration rate of several types of immune cells in the tumor microenvironment (TIME) of HCC. The high glycolysis score group demonstrated statistically significantly lower fractures of CD8 + T cells, CD4 + memory T cells, and regulatory T cells, and higher fractions of helper type1 and type2 T cells and M1 macrophages in the TCGA cohort (Figure 4). However, these results were not validated by GSE76427 cohort except for the result of Th1 cells (*p* = 0.046). Furthermore, there was no association of the score with cytolytic activity score in either cohort. These results suggest that glycolysis signaling level was not associated with immunity in the TIME of HCC.

### 3.5 High glycolysis signaling HCC was associated with worse patient outcomes

**FIGURE 5:**
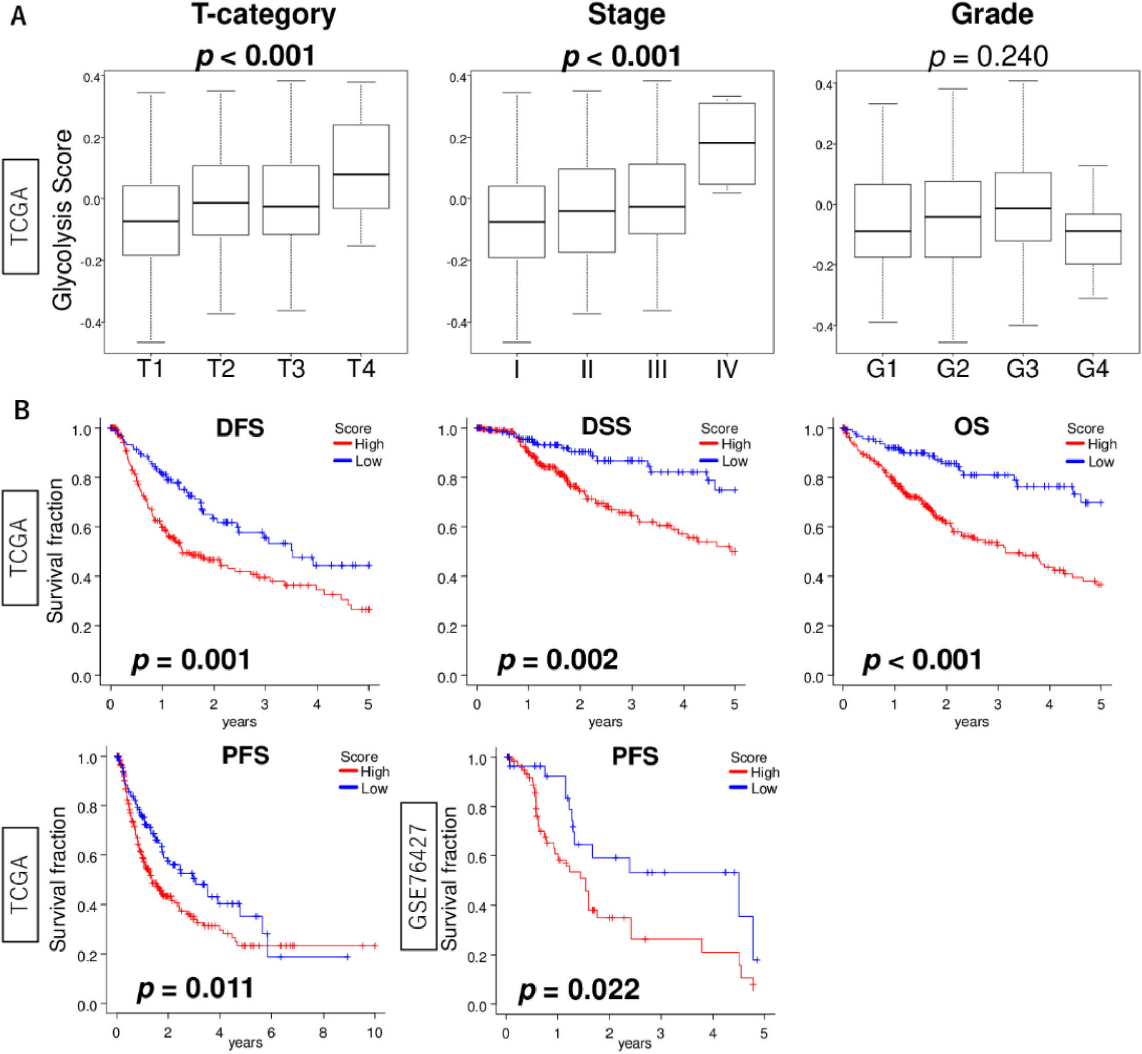
The association of glycolysis signaling score with clinical features of HCC. **(A)** Box plots of glycolysis signaling score level by T stage (T1–T4), AJCC stage (I–IV), and histological grade (G1–G4) in the TCGA cohort. Krustal-Wallis tests were used to investigate for significant differences between groups. **(B)** Kaplan-Meier curves with log-rank *p*-values of DFS, DSS, and OS in the TCGA cohort and PFS in the TCGA and GSE76427 cohorts between HCC with low and high glycolysis signaling scores. AJCC, American joint committee; DFS, disease-free survival; DSS, disease-specific survival; OS, overall survival.

We next investigated the association of glycolysis signaling score levels with clinicopathological features using the TCGA cohort. We found that the score increased with T stage and pathological stage (Figure 5A; both *p* < 0.001). There was no association between the score and tumor grade (*p* = 0.240). We next determined whether HCC with enhanced glycolysis signaling was associated with poorer patient prognosis, and glycolysis signaling score did in fact predict worse outcomes in HCC patients. We found that glycolysis signaling score was associated with survival probability (Figure 5B). Kaplan-Meier survival analysis showed that the high glycolysis score group experienced significantly shorter disease-free survival (DFS), disease-specific survival (DSS), overall survival (OS), and progression-free survival (PFS) compared to the low score group in the TCGA cohort (*p* = 0.001, 0.002, <0.001, and 0.011, respectively). The result for PFS was validated in the GSE76427 cohort (*p* = 0.022). The above suggests enhanced glycolysis signaling in HCC was significantly associated with poor patient survival, and that the glycolysis signaling score could be used for risk stratification of HCC patients.

### 3.6 Glycolysis signaling score was an independent prognostic factor for HCC patients

For the evaluation of possible independent prognostic factors, the following variables were subjected to univariate Cox analysis for OS: age, race, T stage, N stage, M stage, histological grade, and the glycolysis score. In the univariate analysis, T stage, M stage, and the glycolysis score remained as prognostic factors for HCC patients (Table 1). When these results were subsequently subjected to a multivariate Cox analysis, we found that T stage and the glycolysis signaling score were independent, poor prognostic factors of patients with HCC. These results of multivariate analysis indicated that the glycolysis signaling score was an independent predictor of OS as well as DSS for patients with HCC.

**TABLE 1:** Uni- and multivariate analysis in HCC with overall and disease-specific survival in the TCGA cohort.

		**OS**					
		**Univariate**			**Multivariate**		
**Factors**	**Category**	**HR**	**95% CI**	***P*-value**	**HR**	**95% CI**	***P*-value**
Age		1.01	0.99–1.03	0.097			
Race	CA vs. others	1.24	0.87–1.76	0.238			
T	T3/4 vs. T1/2	2.49	1.75–3.55	<0.001*	2.43	157–3.77	<0.001*
N	N+ vs. N−	2.01	0.49–8.19	0.332			
M	M+ vs. M−	3.92	1.23–12.49	0.021*	1.39	0.42–4.65	0.59
Grade	G3/4 vs. G1/2	1.11	0.78–1.58	0.565			
Glycolysis score		15.54	5.64–42.81	<0.001*	6.75	1.92–23.75	<0.001*
		**DSS**					
		**Univariate**			**Multivariate**		
**Factors**	**Category**	**HR**	**95% CI**	***P*-value**	**HR**	**95% CI**	***P*-value**
Age		1.00	0.99–1.02	0.716			
Race	CA vs. others	1.57	0.99–2.48	0.057			
T	T3/4 vs. T1/2	3.44	2.20–5.37	<0.001*	3.44	1.95–6.06	<0.001*
N	N+ vs. N−	3.47	0.84–14.39	0.087			
M	M+ vs. M−	4.96	1.20–20.55	0.028*	1.47	0.33–6.46	0.611
Grade	G3/4 vs. G1/2	1.15	0.73–1.80	0.554			
Glycolysis score		13.55	3.68–49.93	<0.001*	6.56	1.24–34.67	0.027*

CI, Confidence Interval; HR, hazard ratio; OS, overall survival; DSS, disease-specific survival; CA, Caucasian. *Indicates a statistically significant difference.

## 4 Discussion

This study investigated enhanced glycolysis signaling in liver tissue samples across various liver diseases, particularly HCC. While the glycolysis signaling scores did not differ between HCC and other liver diseases, high glycolysis signaling was consistently linked to cell proliferation-related and other pro-cancerous gene sets. This is supported by recent findings that glycolytic enzymes, such as enolase-1 (ENO1) exhibit moonlighting functions that actively contribute to tumor progression. Specifically, ENO1 promotes choline phospholipid metabolism by stabilizing choline kinase-α, thereby supporting cancer cell proliferation (36). HCC with high glycolysis signaling had more homologous recombination defects, intratumor heterogeneity, and mutation rates. No significant associations with immune cell infiltration were found, except for Th1 cells, consistently in both cohorts. Higher glycolysis signaling scores correlated with advanced disease stage and poorer survival outcomes, making the score as an independent prognostic biomarker for HCC.

High glycolysis signaling was also associated with increased homologous recombination deficiency, intratumoral heterogeneity, and elevated mutation burden. These findings suggest that tumors with heightened glycolytic activity may experience greater genomic instability, potentially driven by increased oxidative stress and disruptions in redox homeostasis. Consistent with this notion, pathway analysis demonstrated that high-glycolysis tumors were enriched for gene sets related to the unfolded protein response (UPR), reactive oxygen species (ROS), and DNA repair. These enriched pathways reflect compensatory mechanisms activated in response to metabolic stress and accumulated DNA damage. In particular, upregulation of ROS- and UPR-associated signaling may exacerbate genomic instability and contribute to impaired DNA repair capacity, further promoting mutagenesis. Collectively, these results support a model in which metabolic reprogramming facilitates a cellular environment permissive to genetic alterations and therapy resistance (37, 38).

Due to the heterogeneity of tumor cells, not all of them rely on glycolysis as their main energy source. Glycolysis contributes 2%–64% of total ATP production in tumor cells. Suganuma et al studied the metabolism of four leukemia cell types using the glycolysis inhibitor 2-deoxy-D-glucose (2-DG) and the OXPHOS inhibitor oligomycin (39). They found that NB4 cells were more sensitive to 2-DG, indicating they depend on glycolysis, while THP-1 cells were resistant to 2-DG but sensitive to oligomycin, indicating a reliance on OXPHOS. These findings suggest that the primary energy metabolism pathway varies among tumors. Therefore, to maximize the effectiveness of antimetabolite treatments, it is crucial to understand the metabolic characteristics of each patient’s tumor cells. However, it is difficult to estimate the enhanced level of glycolysis signaling in cancer cells because of their inherent, high heterogeneity (40, 41). Yu Pan et al. developed an aerobic glycolysis index (AGI) to quantify glycolysis activity in HCC, demonstrating that high AGI correlated with poor tumor differentiation, advanced disease stage, and resistance to sorafenib treatment (42).

Understanding the activation level of glycolysis signaling in HCC is crucial for diagnosis, prognostication, and development of treatment strategies. Therapies targeting glycolytic enzymes and metabolites offer promising new treatments, potentially overcoming treatment resistance and enhancing treatment efficacy. Further elucidation of glycolysis mechanisms and advancements in clinical research hold promise for novel approaches to HCC treatment. In the future, investigating the correlation between the therapeutic response of glycolytic drugs on HCC and this scoring system will be necessary. Our study revealed not only the relationship between glycolysis signaling activity and patient prognosis but also the relationship with various underlying factors, such as mutations and the tumor microenvironment (42). We found that there was no significant correlation between the level of glycolysis signaling and the infiltration fraction of immune cells in the TIME, except for Th1 cells. Th1 cells secrete IFN-γ, which activates macrophages and elicits a robust antitumor immune response (43). In HCC, infiltration of Th1 cells is generally associated with a favorable prognosis, as they help suppress tumor growth (44). On the other hand, there are also reports that the ratio of Th2 cells and Treg cells affects prognosis (45). Thus, various factors in the TIME interact in complex ways and affect patient outcomes, so it is important to understand the whole picture rather than focusing on a single factor. Our study method is useful for understanding the overall picture of cancer, and glycolysis signaling activity has been shown to correlate with patient prognosis as a counterbalance to other factors.

Given its association with poor prognosis, genomic instability, and tumor aggressiveness, glycolysis signaling activity may serve as a robust prognostic indicator in HCC. Therapeutic strategies targeting glycolytic enzymes may be particularly effective in patients with high glycolysis scores. Future studies should explore the predictive value of glycolysis scoring in guiding treatment decisions and developing combination therapies to overcome metabolic resistance. However, this study is subject to several limitations. Firstly, its retrospective nature and reliance on public databases introduce the potential for selection bias attributed to the absence of comprehensive clinical data and treatment specifics. This was countered as much as possible through validation of results in completely independent cohorts. Secondly, this study’s observational nature restricts the depth of mechanistic insights, offering merely a static snapshot at a singular time point. We underscore the necessity for additional investigations utilizing preclinical models to elucidate this intricate underlying mechanism comprehensively in future studies. Further research into the detailed mechanisms of glycolysis and their clinical application will open up new perspectives in the treatment of HCC.

## 5 Conclusion

High glycolysis signaling was associated with enhanced level of pro-cancerous and cell proliferation-related gene sets, high levels of homologous recombination defects, intratumor heterogeneity, and mutation rates, and advanced disease stage and poorer survival, making it an independent prognostic factor for HCC.

## Data Availability

The datasets presented in this study can be found in online repositories. The names of the repository/repositories and accession number(s) can be found below: All of these datasets are publicly available without restriction through the Gene Expression Omnibus (https://www.ncbi.nlm.nih.gov/geo/) or cBioPortal (https://www.cbioportal.org/). Accession numbers: GSE6764 (Gene Expression Omnibus), GSE76427 (Gene Expression Omnibus), GSE89377 (Gene Expression Omnibus) and TCGA (via cBioPortal).
